# Spontaneous MEG activity of the cerebral cortex during eyes closed and open discriminates Alzheimer’s disease from cognitively normal older adults

**DOI:** 10.1038/s41598-020-66034-5

**Published:** 2020-06-04

**Authors:** Yoshihisa Ikeda, Mitsuru Kikuchi, Moeko Noguchi-Shinohara, Kazuo Iwasa, Masafumi Kameya, Tetsu Hirosawa, Mitsuhiro Yoshita, Kenjiro Ono, Miharu Samuraki-Yokohama, Masahito Yamada

**Affiliations:** 1 Department of Neurology and Neurobiology of Aging, Kanazawa University Graduate School of Medical Sciences, Kanazawa, Japan; 2 Department of Psychiatry and Neurobiology, Kanazawa University Graduate School of Medical Sciences, Kanazawa, Japan; 3 Department of Preemptive Medicine for Dementia, Kanazawa University Graduate School of Medical Sciences, Kanazawa, Japan; 4 Department of Neurology, NHO Hokuriku National Hospital, Nanto, Japan

**Keywords:** Neuroscience, Diseases, Medical research, Neurology

## Abstract

This study aimed to examine whether magnetoencephalography (MEG) is useful to detect early stage Alzheimer’s disease (AD). We analyzed MEG data from the early stage AD group (n = 20; 6 with mild cognitive impairment due to AD and 14 with AD dementia) and cognitively normal control group (NC, n = 27). MEG was recorded during resting eyes closed (EC) and eyes open (EO), and the following 6 values for each of 5 bands (θ1: 4-6, θ2: 6-8, α1: 8-10, α2: 10-13, β: 13-20 Hz) in the cerebral 68 regions were compared between the groups: (1) absolute power during EC and (2) EO, (3) whole cerebral normalization (WCN) power during EC and (4) EO, (5) difference of the absolute powers between the EC and EO conditions (the EC-EO difference), and (6) WCN value of the EC-EO difference. We found significant differences between the groups in the WCN powers during the EO condition, and the EC-EO differences. Using a Support Vector Machine classifier, a discrimination accuracy of 83% was obtained and an AUC in an ROC analysis was 0.91. This study demonstrates that MEG during resting EC and EO is useful in discriminating between early stage AD and NC.

## Introduction

The most significant risk factor for Alzheimer’s disease (AD) is increasing age^1^. The method used to diagnose AD especially in its early stage needs to be quick and easy such that it can be conducted on many people who have reached a certain age. The measurement of spontaneous brain activity at rest using magnetoencephalography (MEG) could be one of the effective diagnostic procedures for AD, requiring minimum effort from elderly persons.

MEG is a device capable of measuring brain activity easily, non-invasively, and directly. Of several devices that can measure brain function, MEG has the highest temporal resolution using the millisecond time scale. This makes it possible to measure and quantify periodic neuronal activity for each frequency. Compared to electroencephalography (EEG), MEG is not distorted by the resistive properties of the cerebral fluid, skull, and skin, which is an advantage for source-level analysis. While sensor-level analysis applies MEG signals on sensors directly in a statistical or connectivity analysis, source-level analysis estimates the original signal source activities of MEG using a whole-brain model from a magnetic resonance imaging (MRI). Therefore, source-level analysis enables the statistical analysis of each precise brain region^2^.

Human brain networks dynamically switch between eyes closed (EC) and eyes open (EO) resting conditions^3^. Therefore, the resting state choice, either EC or EO, is an essential factor to be carefully considered in brain research^4,5^.

In this study, for early stage AD and cognitively normal control (NC) subjects, we calculated absolute MEG power values during the EC and EO resting conditions for each frequency band in the whole cerebrum and in regions in the Desikan-Killiany atlas^6^ at the source-level on the individual head model using Tikhonov-regularized minimum-norm estimates^7^ from MEG/MRI co-registration data. Additionally, in order to overcome the inter-individual variability in the absolute spectral power, we adopted a whole cerebral normalization algorithm for the estimated regional absolute power values by normalizing them to the whole cerebral power, termed ‘WCN power’. We also examined the absolute power difference between the EC and EO conditions, termed ‘the EC-EO difference’. Finally, we calculated WCN value for the EC-EO difference. These 6 values for 5 frequency bands (θ1: 4–6, θ2: 6–8, α1: 8–10, α2: 10–13, β: 13–20 Hz) in the 68 cerebral regions were compared between the AD and NC groups using a Support Vector Machine (SVM) classifier. MEG analysis using our method would provide suitable clinical indices to differentiate between relatively early stage AD and NC.

## Results

### Characteristics of the AD patients and NC subjects

Twenty AD patients participated in this study. This included 6 with mild cognitive impairment (MCI) due to AD and 14 with probable AD dementia; 27 NC subjects participated in this study. Subject characteristics of the AD and NC groups are shown in Table 1. The AD and NC groups were not significantly different in terms of age, gender, or education level. Mini-Mental State Examination (MMSE)^8^ scores were significantly lower in the AD group than in the NC group. Of the 6 patients with MCI due to AD, 3 were diagnosed with a high likelihood and 3 with an intermediate likelihood^9^. Of the 14 patients with probable AD dementia, 3 were diagnosed with high evidence of the AD pathophysiological process and 11 with intermediate evidence^10^. All of the MCI patients except one were converted to AD dementia within 36 months from MEG recording at MCI stage. The clinical data of each subject in the AD group for the diagnosis including Wechsler Memory Scale-Revised (WMS-R)^11^ index scores and other examination findings are summarized in the Supplemental Table.

**Table 1:** Demographic characteristics of the subject groups.

Group	NC	AD	P-values
Number	27	20	
Male/female	10/17	11/9	0.25^a^
Age (years)	73.2 (63–84)	71.6 (53–81)	0.46^b^
Education (years)	10.5 (4–16)	11.0 (8–16)	0.46^b^
MMSE score	28.7 (23–30)	23.2 (15–30)	<0.0001^b^

Abbreviations: NC, Cognitively Normal Control; AD, Alzheimer’s Disease. MMSE, Mini-Mental State Examination score. Except for the first two rows, values are given as mean (range). ^a^Fisher’s exact test. ^b^ANOVA.

### Comparison of the absolute power between the AD and NC groups

**Figure 1:**
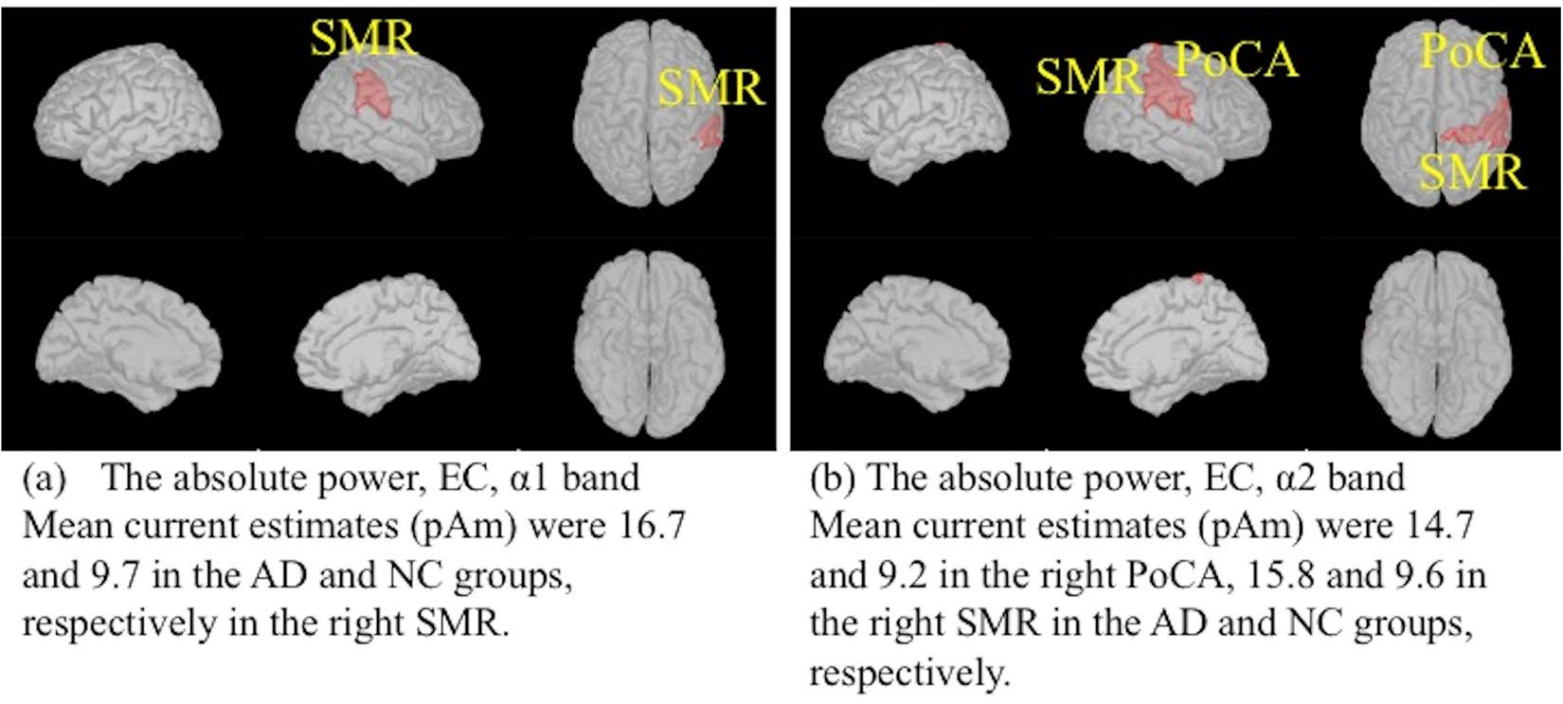
Comparison of the absolute power between the AD and NC groups using two-tailed statistical tests. Regions in the Desikan-Killiany atlas filled with red indicate that the power values in the AD group were higher than those in the NC group. The significance level threshold was 0.000147 (0.05/68 regions/5 frequency bands). (**a**) EC condition, α1 band. (**b**) EC condition, α2 band. PoCA: postcentral area; SMR: supramarginal region. EC: eyes closed. AD: Alzheimer’s disease; NC: cognitively normal control. The figure was drawn using the Brainstorm app (https://neuroimage.usc.edu/brainstorm/Introduction).

During the EC condition, the absolute powers for the α1 band in the right supramarginal region (SMR) (Fig. 1(a)), the α2 band in the right postcentral area (PoCA) and the right SMR (Fig. 1(b)) were significantly higher in the AD group than the NC group. No significant difference between the AD and NC groups was found for any other bands in any other regions during the EC condition. During the EO condition, we could not detect any significant differences in the absolute powers between the AD and NC groups.

### Comparison of the WCN power between the AD and NC groups

**Figure 2:**
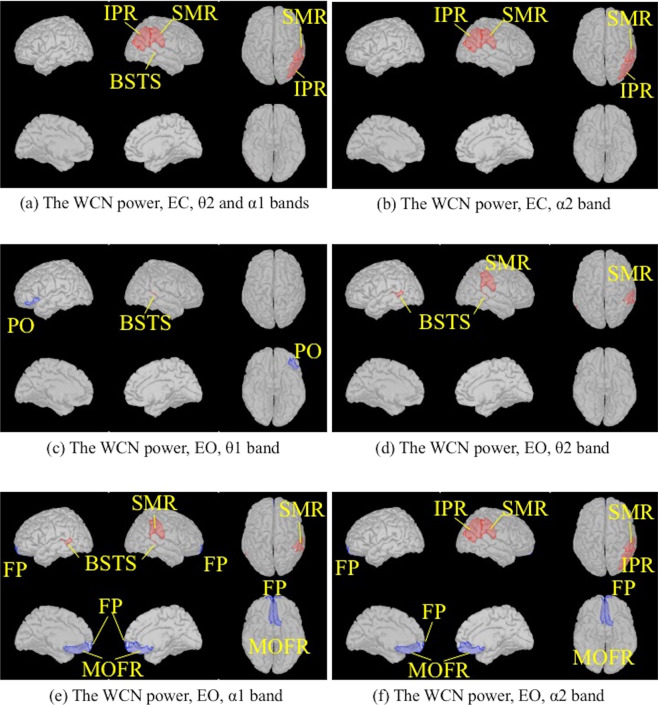
Comparison of the WCN power values between the AD and NC groups using two-tailed statistical tests. Regions in the Desikan-Killiany atlas filled with red indicate that the power values in the AD group were higher than those in the NC group. The significance level threshold was 0.000147 (0.05/68 regions/5 frequency bands). Regions with blue indicate that the power values in the NC group were higher than those in the AD group. (**a**) EC condition, θ2, and α1 bands. (**b**) EC condition, α2 band. (**c**) EO condition, θ1 band. (**d**) EO condition, θ2 band. (**e**) EO condition, α1 band. (**f**) EO condition, α2 band. BSTS: banks of superior temporal sulcus; FP: frontal pole; IPR: inferior parietal region; MOFR: medial orbitofrontal region; PO: pars orbitalis; SMR: supramarginal region. EC: eyes closed; EO: eyes open. AD: Alzheimer’s disease; NC: cognitively normal control. The figure was drawn using the Brainstorm app (https://neuroimage.usc.edu/brainstorm/Introduction).

The WCN powers in the AD group during the EC condition were higher than those in the NC group for the θ2 and α1 bands in the right banks of the superior temporal sulcus (BSTS), the right SMR and the right inferior parietal regions (IPR) (Fig. 2(a)), and for the α2 band in the right SMR and IPR (Fig. 2(b)). We could not find any significant difference between the AD and NC groups for any other bands in any other regions. During the EO condition, the WCN powers in the AD group were higher than those in the NC group for the following bands: the θ1 band in the right BSTS (Fig. 2(c)), θ2 band in the bilateral BSTSs and the right SMR (Fig. 2(d)), α1 band in the bilateral BSTSs and the right SMR (Fig. 2(e)), α2 band in the right SMR and IPR (Fig. 2(f)). Conversely, the WCN powers in the AD group were lower than those in the NC group for the α1 and α2 bands in the bilateral medial orbitofrontal regions (MOFRs) (Fig. 2(e,f)). We could not find any significant differences between the AD and NC groups for any other bands in any other regions.

### Comparison of the EC-EO difference between the AD and NC groups

**Figure 3:**
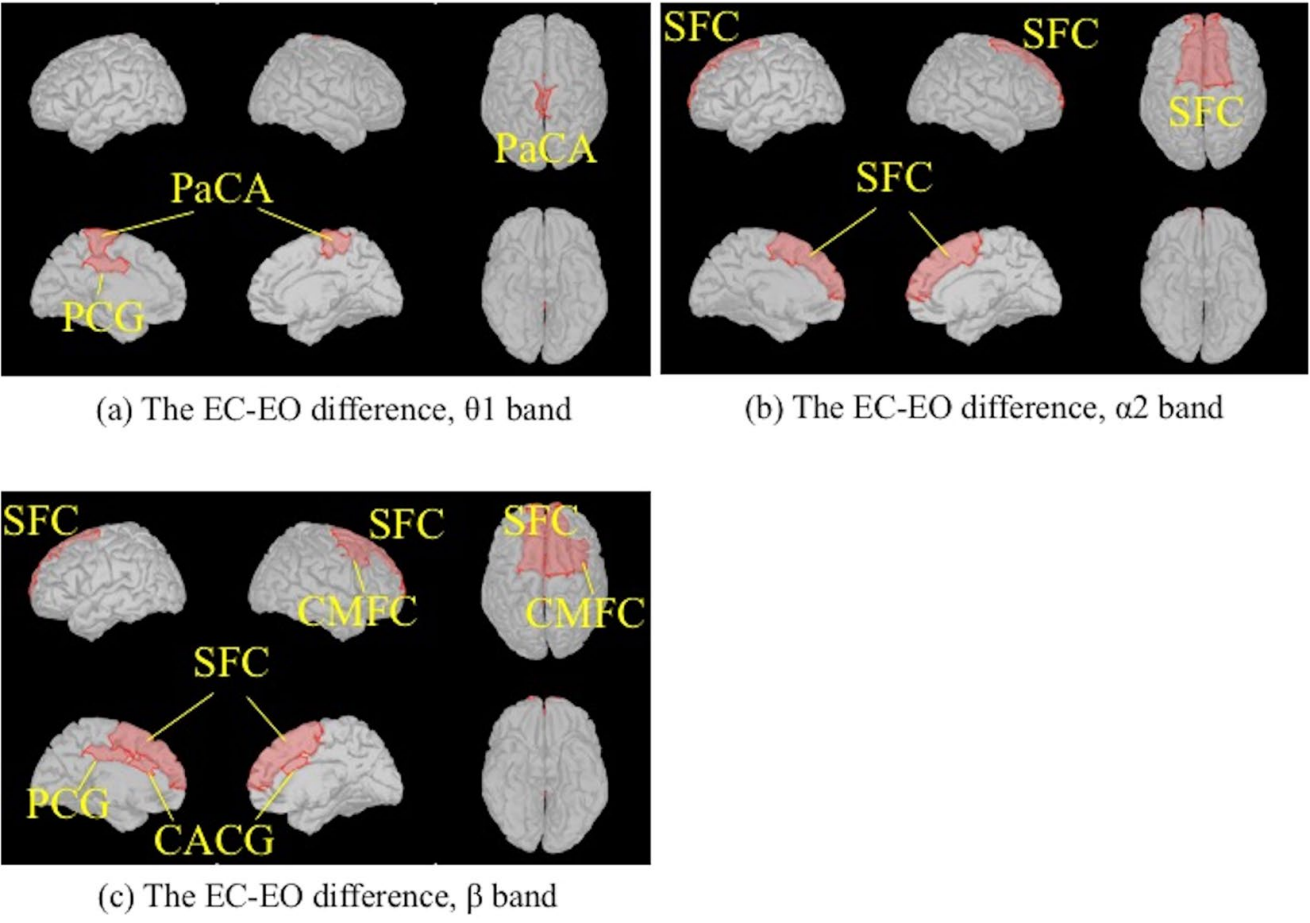
Comparison of the EC-EO differences between the AD and NC groups using two-tailed statistical tests. Regions in the Desikan-Killiany atlas filled with red indicate that the differences in the AD group were higher than those in the NC group. The significance level threshold was 0.000147 (0.05/68 regions/5 frequency bands). (**a**) θ1 band. (**b**) α2 band. (**c**) β band. CACG: caudal anterior cingulate gyrus; CMFC: caudal middle frontal cortex; PaCA: paracentral area; PCG: posterior cingulate gyrus; SFC: superior frontal cortex. EC: eyes closed; EO: eyes open. AD: Alzheimer's disease; NC: cognitively normal control. The figure was drawn using the Brainstorm app (https://neuroimage.usc.edu/brainstorm/Introduction).

The absolute power was generally higher during the EC condition than the EO condition in both the AD and NC groups. The EC-EO differences were higher in the AD group than those in the NC group for the θ1 band in the left posterior cingulate gyrus (PCG), the bilateral paracentral areas (PaCA) (Fig. 3(a)), the α2 band in the bilateral superior frontal cortices (SFCs) (Fig. 3(b)), the β band in the bilateral SFCs, the left PCG, the bilateral caudal anterior cingulate gyri (CACGs) and the right caudal middle frontal cortex (CMFC) (Fig. 3(c)). There were no significant variations seen in the EC-EO differences between the AD and NC groups for any other bands in any other regions.

### Comparison of the WCN value for EC-EO difference between the AD and NC groups

We could not find any significant difference between the AD and NC groups for the WCN value for the EC-EO difference for any bands in any regions.

### Discrimination accuracy between the AD and NC groups

**Figure 4:**
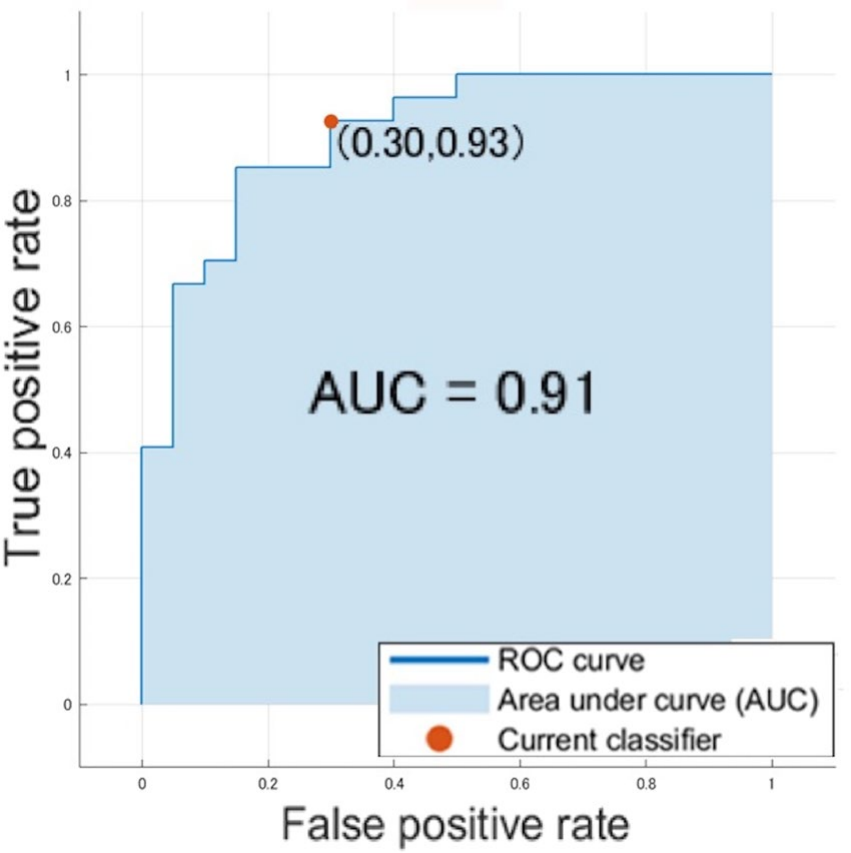
ROC analysis of the datasets by a support vector machine classifier.

By the datasets of the 2,040 parameters (=6 values × 5 frequency bands × 68 regions) per person mentioned above, a discrimination accuracy of 83% (sensitivity 70% and specificity 93%) was obtained and an AUC in an ROC analysis was 0.91 (Fig. 4) using a SVM classifier.

## Discussion

The majority of the previous studies for patients with AD using EEG or sensor-level MEG were reported under the EC resting condition. Many of these studies asserted that, in the occipital, temporal, and parietal areas, α and β activities are lower and θ and δ activities are higher in patients with AD than in NC^5,14–17^. Under the EO condition, some studies on EEG in patients with AD have also described a decrease in α power and increase in θ power^18–20^. In the MEG analysis under the EC condition, we revealed that the AD group had higher absolute α band powers in the SMR and the PoCA as compared to the NC group. Also, during the EC and EO conditions, the AD group had higher WCN powers for the θ2 − α band in the BSTS and the SMR as compared to the NC group. Similar findings in source-level MEG studies have shown that AD patients had predominant θ − α band sources in the temporal regions as compared to NC subjects^12,13^. Previous EEG reports have shown that α activity is inversely related to cortical activity^21,22^. The pathological changes of AD in the brain generally start in the posterior-temporal regions^23^. A fluoro-2-deoxy-D-glucose positron emission tomography imaging study revealed that glucose metabolic reductions in the parieto-temporal cortices are a key sign of early stage AD^24^. The BSTS and SMR are quite close to the posterior-temporal and parieto-temporal regions. These regions may help to indicate the region of cortical hypo-activity in early stage AD. MEG could assist in detecting the hypo-activity lesions in AD patients.

In the AD group, the WCN power values for the α band in the bilateral FPs and the MOFRs were lower than in the NC group. A study using EEG and functional MRI has reported a strong negative correlation of frontal cortical activity with α band power^22^. These findings might suggest that patients with AD have cortical hyperactivity in the frontal areas of the brain. Functional MRI study has shown greater activations in AD patients relative to NC subjects, particularly in the frontal cortex^25^. A study using Tc-99m hexamethylpropyleneamine oxime also demonstrated that patients with progressive MCI showed higher regional cerebral blood flow in the prefrontal cortex as compared to NC subjects^26^. The WCN power values for the α band of MEG might detect characteristics of cerebral frontal cortical activity in patients with AD.

A few previous EEG studies comparing EC and EO resting conditions have demonstrated reduced reactivity of α band power in patients with AD compared with the NC^14,27,28^. Using sensor-level frequency analysis, previous MEG studies have reported that the difference in α band powers between the EC and EO conditions in posterior areas were smaller in AD than in NC subjects^29,30^. However, in this study using source-level analysis, the EC-EO differences were significantly larger in the AD patients than in the NC subjects for the θ1 band in the bilateral PaCAs, for the α2 and β bands in the bilateral SFCs, and for the β band in the bilateral cingulate gyri. Consistent with our results, previous studies that have used source-level frequency analysis have reported that the power ratio of the EC to EO condition in the 8–15 Hz band in the dorsal frontal regions in patients with AD was significantly larger than that in NC^31,32^. Intriguingly, the topological organization of the human brain networks was thought to dynamically switch corresponding to the information processing modes of the EO or EC conditions^3^. Therefore, this study suggests that the dorsal frontal, paracentral, and cingulate regions might be involved in visual dynamic nodal properties of networks and that these networks might be impaired in AD. A disturbed brain network is a distinctive feature of AD^33,34^. This might contribute to larger power differences in those regions between the EC and EO conditions in patients with AD as compared to NC subjects.

Previous EEG studies explored the abilities of several classification algorithms including SVM, feature selection and others to discriminate AD patients from healthy controls^35,36^. In this study, we attempted the classification with the cerebral regional MEG activities through the source-level analysis and presumed to have achieved a certain predictive performance for early stage AD as mentioned above.

This study has some limitations. Firstly, the number of subjects was small. Secondly, we did not obtain histopathologic verification of the AD diagnosis for any patient. Thirdly, further studies with various types of dementia are necessary to conclude whether the MEG findings in this study are specific for AD.

In conclusion, our results suggest that MEG recording during EC and EO conditions at rest using individual head model to detect cortical activities that express the brain networks of visual information would be useful in evaluating cortical functional aspects of early stage AD. It will also be useful in discriminating between AD and NC.

## Methods

### Subjects

We recruited patients diagnosed as having probable AD dementia with an intermediate or high evidence of the pathophysiological processes of AD or MCI with an intermediate or high likelihood of AD, according to the diagnostic guidelines for Alzheimer’s disease from the National Institute on Aging-Alzheimer’s Association^9,10^. All patients were examined by neurologists, and we excluded patients receiving medications acting upon the central nervous system (i.e. cholinesterase inhibitors, N-Methyl-d-aspartate receptor antagonists, antipsychotics, anticholinergics, antidepressants, anticonvulsants, benzodiazepines, cerebral metabolic activators, or cerebral vasodilators). Blood tests and an MRI of the brain were performed to eliminate any other potential medical conditions. NC volunteers were recruited from participants in the Nakajima study. This is a population-based longitudinal cohort study that investigated cognitive decline in residents aged 60 years or older in Nakajima, Ishikawa Prefecture, Japan^37^. The NC group had no history of psychiatric or neurological diseases and were receiving no medications acting upon the central nervous system. All were assessed as cognitively normal. Cognitive profiles were evaluated using the MMSE, and the Japanese translation of WMS-R. This study was conducted according to the guidelines of the Declaration of Helsinki and all procedures involving human subjects were approved by the Kanazawa University Medical Ethics Review Board (approval number 699). Written informed consent was obtained from all subjects or their legal representatives.

### MEG recordings and MRI scans

MEG measurements were performed using a MEG system (PQA160C, Yokogawa Electric Corporation, Kanazawa, Japan). The system consisted of a 160-channel whole-head coaxial gradiometer. MEG signals were processed through an on-line bandpass filter of 0.25–500 Hz with a digital sampling rate of 1,000 Hz. In a magnetically shielded room, the position of the head within the helmet was determined by measuring the magnetic fields after passing currents through coils that were attached at 5 locations on the surface of the head as fiduciary points to the landmarks (nasion and pre-auricular points). Two minutes of spontaneous MEG activity were recorded with subjects relaxed in the supine position on a bed under the EC and EO conditions. In the EO condition, the subjects were asked to look at a small point projected on a screen about 30 cm above their faces. Their faces and upper bodies were tracked using a camera, and their live MEG waves were monitored to show their wakefulness. Using the Sigma Excite HD 1.5 T system (GE Yokogawa), all subjects underwent T1-weighted MRI studies. To superimpose the coordinate system of MEG on the MRI images^38^, T1-weighted MRI was performed with spherical lipid markers placed at the five MEG fiduciary points. These MRI images consisted of 158 sequential horizontal slices of 1.2 mm thickness, with a resolution of 512 × 512 points in a field of view of 261 × 261 mm.

### Data processing

The MEG waves contaminated by large-amplitude noise exceeding 10 pT were removed using the analysis software MEG Laboratory (Yokogawa Electric Corporation, Kanazawa, Japan). This was attached to the MEG device to obtain data files of 112–120 seconds. Using the analysis software FieldTrip, coarse artifacts were removed through a principal component analysis and heartbeat component as well as ophthalmic and palpebral electromyograms were removed through an independent component analysis^39^. Source reconstruction was performed using Brainstorm^2^, which was available online for downloading using the GNU general public license. We applied the band definition of a study which analyzed spontaneous MEG activity^16^. An overview of MEG power spectral in higher frequency than 20 Hz (i.e., β2 and γ bands) seemed to show not much difference between the AD and NC groups. Besides, our MEG recordings had much artifacts in 1–4 Hz (i.e., δ band) that were difficult to be removed separately. Hence, we decided 4–20 Hz (i.e., θ, α, β1 bands) as the analysis object. Finally, we divided θ and α bands into sub-bands expecting improvement of discrimination accuracy between the groups, and defined them as θ1 (4–6 Hz), θ2 (6–8 Hz), α1 (8–10 Hz), α2 (10–13 Hz), and β (13–20 Hz) bands. The data files were divided into 55–59 segments of 2 seconds and were separated into 5 bands through a bandpass filter. To estimate the brain source, an anatomically constrained MEG approach was used that places an anatomical constraint on the estimated source by assuming that an individual’s recorded brain activity lies in the cortical mantle^40^. Landmark information and digitized head surface points for MEG/MRI co-registration were used. Sensors were registered for each individual with a fiducial landmark using FASTRAK (Polhemus, VT, USA). The digitized head shape and the scalp surface of each individual were then used to reduce the minimum distance error between them in an iterative process. Cortical surfaces were created for each individual by automatically segmenting the T1-weighted MRIs into gray and white matter. The border between the gray and white matter was then defined as the cortical surface. Cortical reconstruction of the MRI data and volumetric segmentation were performed in FreeSurfer^41^. The lead field was then computed using the overlapping spheres algorithm^42^ with a cortical surface tessellated with 15,000 vertices. The inverse solution was calculated for each individual using Tikhonov-regularized minimum-norm estimates^7^. Therefore, a weighted minimum-norm estimation with source orientation constraints was chosen to compute the source activity for all of the 2-second segments. The absolute values of the source activities at all-time points (55–59 segments × 2 seconds × 1000 Hz sampling rate) were averaged on each vertex and in each frequency band, using the ‘average files’ and the ‘average time’ process. Finally, current estimates as ‘absolute powers’ of regions provided in Brainstorm, which contain many vertices in the atlases, could be obtained using the ‘scouts time-series’ process. We used the Desikan-Killiany atlas^6^ equipped in Brainstorm, which divided the cerebral cortex into 68 regions symmetrically. In addition, we proposed a whole cerebral normalization algorithm for the estimated source or cortex level power values to overcome the inter-individual variability in absolute spectral power^43^. This was done by normalizing the power in a reference region to the whole cerebral power. In this study, we have termed this normalization as whole cerebral normalization (WCN). WCN power in region *a* was calculated as the z-score using the following formula: [(absolute power in region *a*) − (whole cerebral mean absolute power)]/(standard deviation of absolute powers in all regions). We also calculated the power difference by subtracting the source localized absolute power during the EO condition from that during the EC condition. We have termed this ‘the EC-EO difference’. Finally, we calculated WCN value for the EC-EO difference.

### Statistical analysis

The following values were compared between the groups for the 5 frequency bands in the 68 regions: (1) the absolute power during EC and (2) EO, (3) the WCN power during EC and (4) EO, (5) the EC-EO difference, and (6) WCN value for the EC-EO difference. The *p*-value was based on a two-tailed statistical test with a significance level threshold of 0.000147 calculated as: 0.05 / 68 regions in the Desikan-Killiany atlas / 5 frequency bands. We trained a support vector machine (SVM) to classify the data described above as NC or AD, and computed the accuracy scores using the observations in the six validation folds and reported the average cross-validation error. It also made predictions on the observations in these validation folds and computes the confusion matrix and ROC curve based on these predictions.

### Support vector machines

SVM is a supervised learning algorithm that can be used for binary classification or regression. A support vector machine constructs an optimal hyperplane as a decision surface such that the margin of separation between the two classes in the data is maximized. Support vectors refer to a small subset of the training observations that are used as support for the optimal location of the decision surface. Hence, it is inherently applicable for smaller data sets^44^. Firstly, we separated randomly the data into training (n = 1700) and test sets (n = 340). Each set contains the class label (i.e., NC or AD) and features (i.e., theta power at each brain region). Then, the SVM was trained on the training set so that it was able to predict the class label of the test sets based on the features of those. Training for a SVM has two phases: (1) transform predictors (input data) to a high-dimensional feature space. It is sufficient to just specify the Kernel for this step and the data is never explicitly transformed to the feature space (i.e., Kernel trick). Here, we used second-order polynomials as Kernel functions as it well performed on the datasets. Then, (2) solve a quadratic optimization problem to fit an optimal hyperplane to classify the transformed features into two classes. The number of transformed features is determined by the number of support vectors. Only the support vectors chosen from the training data are required to construct the decision surface. Once trained, the rest of the training data are irrelevant. We repeated this procedure six times to assess how accurately the obtained SVMs will perform on the test sets (cross-validation). The purpose of this step was to test the SVM’s ability to predict new data that was not used in estimating it, in order to overcome problems like overfitting or selection bias and to give an insight on how the model will generalize to an independent dataset. For further details and validity of SVM algorithms can be found elsewhere^45–48^. The procedure was done with MATLAB and the Classification Learner app.

## Supplementary information

Supplementary information.

## Data Availability

The datasets generated and/or analyzed during the current study are available from the corresponding author on reasonable request.
