# Supplementary material for: Spontaneous MEG activity of the cerebral cortex during eyes closed and open discriminates Alzheimer’s disease from cognitively normal older adults
Source: Sci Rep. 2020 Jun 4;10:9132. doi: 10.1038/s41598-020-66034-5 (PMC7272642; doi:10.1038/s41598-020-66034-5)
Supplement: Supplementary file 1 — Supplementary information. [file 41598_2020_66034_MOESM1_ESM.pdf]

Spontaneous MEG activity of the cerebral cortex during eyes closed and open discriminates Alzheimer's disease from cognitively normal older adults

Yoshihisa Ikeda, Mitsuru Kikuchi, Moeko Noguchi-Shinohara, Kazuo Iwasa, Masafumi Kameya, Tetsu Hirosawa, Mitsuhiro Yoshita, Kenjiro Ono, Miharu Samuraki-Yokohama and Masahito Yamada.

Supplemental table. The clinical data of subjects in the Alzheimer's disease group.

No	Age	Gender	MMSE	WMS-R*					CSF		MRI	PET		MCI or dementia at MEG recording	Duration from MEG recording at MCI stage to conversion to dementia (months)
				Verbal memory	Visual memry	General memory	Attention / Concentration	Delayed recall	Aβ42 (pg/ml) (normal, >490)**	ptau (pg/ml) (normal, <49)**	Visual MTA score (pathological atrophy, ≧ 2)***	Site of cerebral Aβ deposits****	Site of cerebral glucose hypometabolism****		
1	53	M	30	55	75	55	105	<50	633	118	2	B PCG, B F, B T	L P, L T, B PreC, B PCG, L O	MCI	22
2	56	M	28	92	64	80	101	55	255	47	2	B PCG, R T, B F	B CG, B PreC, B P-T, B T	MCI	31
3	60	F	27	60	70	57	102	<50	NT	NT	1	B PCG, B F, B P, BT	L PreC, L P	MCI	25
4	65	F	25	67	100	77	114	51	939	98	1	NT	L PCG, B O	MCI	6
5	69	F	27	72	60	64	UA	50	NT	NT	2	NT	B PCG, R PreC	MCI	36
6	72	M	28	98	94	96	99	79	NT	NT	2	NT	R P, R F, L PCG	MCI	>72
7	68	M	26	68	95	74	81	<50	503	82	1	NT	NT	dementia	-
8	68	M	23	111	90	104	85	75	368	83	3	NT	NT	dementia	-
9	69	M	20	79	90	80	84	63	NT	NT	3	NT	B PCG, R PreC, R F, R P, R T	dementia	-
10	70	F	21	55	94	67	99	59	404	81	2	NT	NT	dementia	-
11	73	M	22	64	63	61	102	57	NT	NT	3	NT	NT	dementia	-
12	75	M	26	99	91	94	99	81	NT	NT	2	NT	NT	dementia	-
13	76	F	15	58	<50	50	68	51	NT	NT	2	NT	B P, L T, L PreC	dementia	-
14	77	M	18	UA	UA	UA	81	UA	NT	NT	3	NT	B P, B PCG, B PreC, B F	dementia	-
15	78	M	28	76	<50	63	95	69	NT	NT	2	NT	NT	dementia	-
16	80	F	25	70	68	67	92	62	NT	NT	2	NT	B F, R P, B PreC, B PCG	dementia	-
17	80	F	19	69	<50	57	63	55	NT	NT	3	NT	B PCG, B PreC, B P, B T	dementia	-
18	80	F	17	53	56	52	89	62	NT	NT	2	NT	NT	dementia	-
19	81	F	21	59	<50	51	92	<50	NT	NT	2	NT	B PreC, B P, B F, R T	dementia	-
20	81	M	18	60	63	59	76	50	358	142	3	NT	B P, B T, B ACG, B PCG	dementia	-

Abbreviations: Aβ, beta-amyloid protein; CSF, cerebrospinal fluid; MCI, mild cognitive impairment; MEG, magnetoencephalography; MMSE, Mini-Mental State Examination score; MRI, magnetic resonance imaging; MTA, medial temporal lobe atrophy; NT, not tested; PET, positron-emission tomography; ptau, phosphorylated-tau; UA, unable to be assessed; WMS-R, Wechsler memory scale-revised index score.

* The WMS-R Japanese version is not normalized for persons aged 75 years and over, so their score were given by taking them as belonging in the 70-74 age group.

** Morinaga, A., et al., Dement Geriatr Cogn Disord, 2010. 30(4).

*** Wahlund, L.O., et al., J Neurol Neurosurg Psychiatry, 2000. 9.

**** ACG, anterior cingulate gyrus; B, bilateral; CG, cingulate gyrus; F, frontal lobe; L, left; O, occipital lobe; P, parietal lobe; PCG, posterior cingulate gyrus; PreC, precuneus; R, right; T, temporal lobe.
